# From Strep Infection to a Strepitous Heart Pattern in Rheumatic Fever: A Case Report

**DOI:** 10.7759/cureus.72997

**Published:** 2024-11-04

**Authors:** Jomar N Machuca

**Affiliations:** 1 Internal Medicine, Veterans Affairs Medical Center, San Juan, PRI

**Keywords:** auto-immune molecular mimicry, echocardiography - heart failure - valvular heart disease, mitral valve stenosis, post strept complications, rheumatic heart diseases

## Abstract

Rheumatic heart disease (RHD) is the leading cause of valvular heart disease globally, arising from acute rheumatic fever (ARF). It results from an abnormal immune response to group A streptococcal (GAS) infection, leading to myocardial injury. This is the case of a 65-year-old female with severe mitral regurgitation (MR) secondary to RHD disease who develops acute heart failure with preserved ejection fraction (HFpEF) and atrial fibrillation (AF), both de novo. It underscores the role of echocardiography in early diagnosis and severity assessment of RHD, antibiotic prophylaxis strategies, and the management of complications.

## Introduction

Rheumatic heart disease (RHD) is a chronic complication of acute rheumatic fever (ARF), characterized by myocardial injury secondary to an abnormal immune response to group A streptococcal (GAS) infection [1]. It leads to valvular stiffening, commissural fusion, and chordae tendineae shortening, resulting in regurgitation and stenosis [2]. According to the Global Burden of Disease, RHD affected approximately 40 million people in 2019 [3], with the highest prevalence in developing regions due to limited healthcare access [2-4]. Oceania, Sub-Saharan Africa, and South Asia have the highest incidence, with emerging cases in industrialized areas due to immigration [2-7]. ARF predominantly affects children and adolescents, while RHD commonly presents later in life, often in women around 28 years old [2,5]. RHD mortality ranges from 260,000 to 300,000, and over 10 million disability-adjusted life-years are lost [2,4,5]. It is now recognized that GAS skin infections can trigger ARF (in addition to streptococcal throat infections) [8]. ARF typically occurs three weeks after GAS infection and affects various organs, including the heart [2,8]. Molecular mimicry, the most common pathophysiological mechanism for RHD, involves cross-reactivity between GAS antigens and cardiac proteins, leading to autoimmunity. GAS antigens such as the M protein and group A carbohydrate trigger anti-endothelial antibody production by immune cells that adhere to the endocardium, promoting T-cell entry and cytokine-mediated valvular injury. Granulomatous heart muscle inflammation mediated by this type IV hypersensitivity reaction against GAS antigens forms the pathognomonic Aschoff bodies [1-7]. The type I collagen exposure caused by valvular damage also triggers autoimmunity, perpetuating the inflammatory response [1,7,8]. The recent neo-antigen theory suggests that changes in collagen structure in the basement membrane, mediated by complex formation between the M protein and the CB3 region of type IV collagen, trigger an antibody response against type IV collagen [1,8]. Recurrent ARF episodes cause progressive fibrosis of heart valves, leading to RHD in about 60% of patients with carditis [2,8,9]. Both pathophysiological explanations are considered potential mechanisms of RHD. The precise reasons for the autoimmunity's preference for cardiac tissue and left-sided heart valves remain under study [2].

## Case presentation

**Figure 1:**
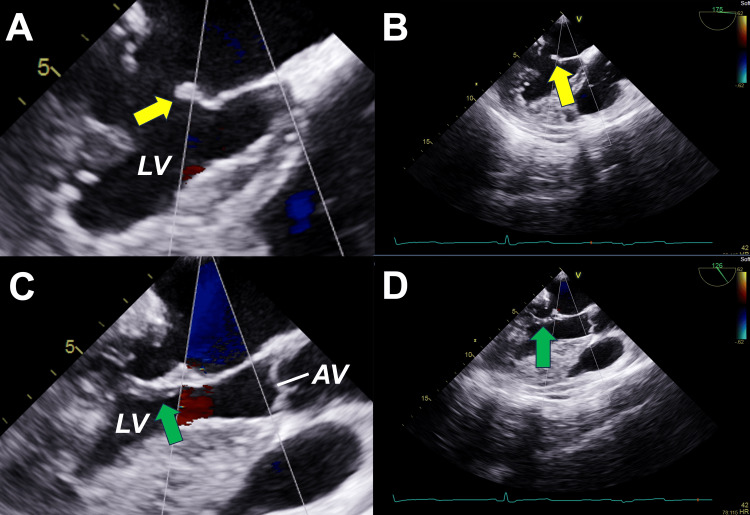
TEE showing rheumatic MV Images show a thickened MV (A-D), the “hockey stick” sign or diastolic doming of the anterior MV leaflet (yellow arrow in A and B), and calcification of the posterior MV leaflet (green arrow in C and D). LV: left ventricle, AV: aortic valve.* * Source: Images are original from the case.

**Figure 2:**
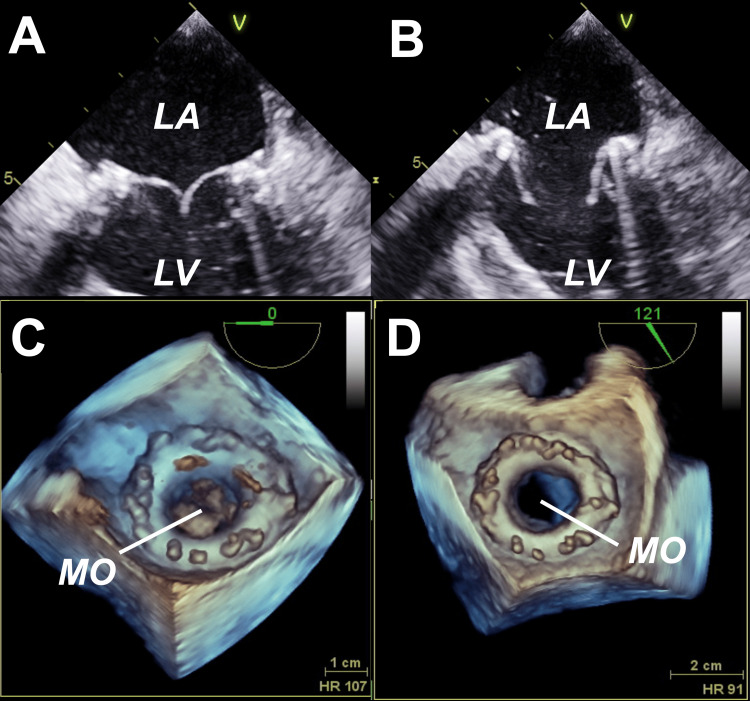
TEE after MV replacement Images show MV replacement with adequate closure in 2D (A and B) and 3D (C and D). LA: left atrium, LV: left ventricle, MO: mitral opening. Source: Images are original from the case.

A 65-year-old Dominican female with hypertension, type 2 diabetes mellitus, obesity class III, chronic obstructive pulmonary disease on home oxygen, and an active smoker presented with a three-day history of progressive shortness of breath, orthopnea, and bilateral leg edema. She reported no chest pain, palpitations, cough, fever, or recent infections. Vital signs showed a temperature of 37.1 °C, blood pressure of 104/52 mm Hg, tachycardia at 108 beats/min, tachypnea at 21 breaths/min, and oxygen saturation of 87% on room air. Physical examination revealed jugular venous distention, an irregular pulse, a holosystolic murmur at the apex, inspiratory crackles, decreased bibasilar breath sounds, and lower extremity edema. The electrocardiogram (ECG) showed atrial fibrillation (AF) with a rapid ventricular response and no acute ischemic changes. Blood tests were remarkable for elevated creatinine levels at 1.37 mg/dL, elevated pro-brain natriuretic peptide at 1219 pg/mL, and negative high-sensitivity cardiac troponin T levels at 4 ng/L (reference <14 ng/L). Chest X-ray showed congestive changes and small bilateral pleural effusions. Chest CT angiogram ruled out pulmonary embolism. The patient was admitted due to acute heart failure with preserved ejection fraction (HFpEF) and AF, both de novo. Transthoracic echocardiogram (TTE) showed preserved left ventricular ejection fraction (LVEF), severe mitral regurgitation (MR), no intracardiac thrombi, and a high estimated pulmonary artery systolic pressure of 55 mmHg. She received IV furosemide, IV amiodarone, and full-dose anticoagulation with enoxaparin. Transesophageal echocardiogram (TEE) revealed an LVEF of 50%, moderate to severe left atrial (LA) dilation, severe MR, an eccentric jet with posterior projection, and a fixed posterior mitral valve (MV) leaflet with calcification (Figure 1). Acute heart failure resolved, and the patient returned to normal sinus rhythm. The patient underwent a left and right heart catheterization for evaluation of ischemic heart disease and pulmonary hypertension (P-HTN). The catheterization evidenced 1-vessel coronary artery disease (left circumflex with 70% obstruction), normal LV end-diastolic pressure at 10-15 mmHg, and mild combined pre- and post-capillary P-HTN with preserved cardiac output and cardiac index. She was discharged home with medical therapy for heart failure (valsartan, metoprolol succinate, and empagliflozin), amiodarone and apixaban for AF, and a high-intensity statin. In preparation for MV replacement, she underwent a dental evaluation. The following week, the MV was surgically replaced with a 27-mm bioprosthetic valve without complications. A post-procedure TEE confirmed normal systolic function, adequate valve replacement, and no thrombus (Figure 2). In addition to the above-mentioned medical therapy, she was discharged home with Aspirin 81 mg for three months. She received continuous RHD secondary prophylaxis with intramuscular Benzathine Penicillin G (BPG) 1.2 million units every four weeks.

## Discussion

ARF can present with arthritis, fever, carditis, subcutaneous nodules, erythema marginatum, and Sydenham chorea [2,7]. Rheumatic carditis, the most common manifestation in the first ARF episode, progresses to RHD in up to 70% of cases [5,7]. While RHD is the primary cause of mitral stenosis (MS) worldwide, younger individuals present more commonly with MR [2]. As in this case, patients with RHD may present with valvulopathies, heart failure, and arrhythmias such as AF. Other presentations include myopericarditis, infective endocarditis, P-HTN, and ischemic stroke [2,4]. RHD primarily affects left-sided valves, especially the mitral valve, in up to 60% of cases, with MR occurring early and MS developing over time [1,2,4,5,9]. The first symptom of RHD, as seen in this case, is usually worsening exertional dyspnea [2,10]. Patients may also experience hemoptysis due to pulmonary congestion, and in rare cases, they may experience chest pain due to right ventricular (RV) ischemia caused by RV hypertrophy in the setting of P-HTN. A physical exam may show signs of volume overload, as evidenced by the case presented. A prominent “a” wave from elevated right atrial pressure in patients with P-HTN may also be seen [2,10]. However, these waves will be absent if the patient has already developed AF by diagnosis. On auscultation, MS is characterized by an opening snap caused by the abrupt tensing of the valve leaflets followed by a diastolic rumble. The onset of the rumble correlates with stenosis severity: as the valve becomes more stenotic, the transvalvular gradient increases, and the rumble is heard earlier in diastole [10,11]. MR often presents with an apical holosystolic murmur [2]. While auscultation can be helpful, it is not sensitive or specific for RHD [5]. An ECG may reveal LA enlargement, which can lead to AF. Atrioventricular (AV) blocks can occur in myocarditis. While third-degree AV blocks are typically temporary, some may be persistent and require a permanent pacemaker [2-4,6]. Advanced cases can show cardiomegaly and pulmonary venous congestion on a chest X-ray [4].

Diagnosis

RHD may remain subclinical, particularly in developing countries, as more than 50% of RHD patients lack a history of ARF [4]. In this case, the patient comes from an RHD-endemic country [12]. She did not have any history of ARF, and the diagnosis of RHD was made after the onset of heart failure symptoms and AF. According to a systematic review and meta-analysis, subclinical RHD is about seven to eight times more common than clinically evident cases, leading to potential underdiagnosis until symptoms appear [2]. Echocardiography significantly improves RHD detection compared to clinical assessment alone, identifying small valvular nodules before the onset of symptomatic disease [1,7]. According to the 2012 World Health Federation guidelines, diagnosis of RHD requires pathological MR or aortic regurgitation plus at least two morphological features of RHD [2,6,7]. The guidelines classify patients into definite RHD, borderline RHD, and normal categories to enhance sensitivity in high RHD-prevalent populations, particularly for younger patients who may not yet exhibit all the features of definite RHD [6,9]. Some studies conducted in RHD-endemic areas suggest the benefit of echocardiography screening in subclinical RHD cases for initiating antibiotic prophylaxis. However, further research is necessary to evaluate its role at the population level before implementing it into a global routine screening program [4-6].

**Figure 3:**
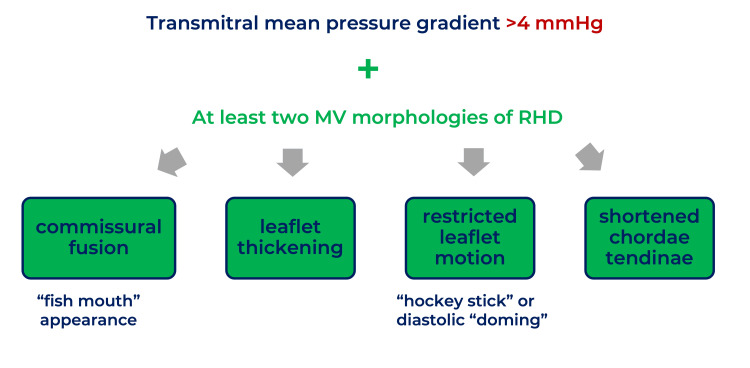
Diagnostic criteria for rheumatic MS This image was created by Dr. Jomar Machuca.

**Figure 4:**
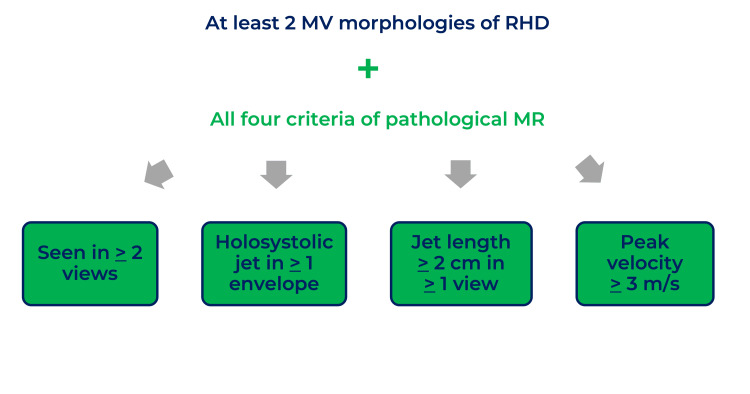
Diagnostic criteria for rheumatic MR This image was created by Dr. Jomar Machuca.

**Figure 5:**
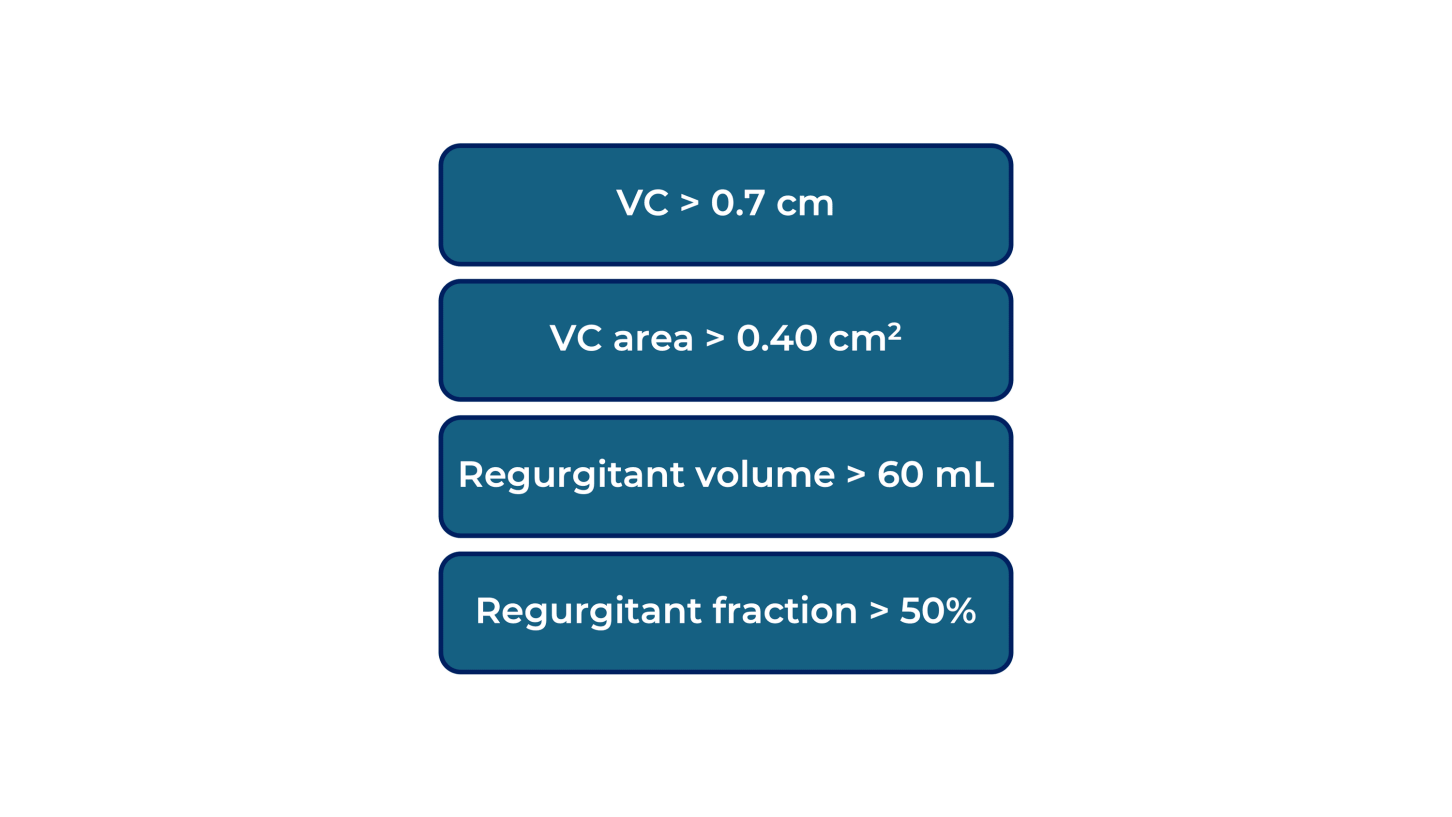
Criteria for severe MR VC: vena contracta. This image was created by Dr. Jomar Machuca.

The MV is commonly involved in RHD. MS is chronic valvulopathy from RHD, diagnosed by a transmitral mean pressure gradient over 4 mmHg and at least two characteristic findings of rheumatic valvulopathy, as detailed in Figure 3 [2,6,7]. On the other hand, MR results from thickened valve leaflets and shortened chordae, causing inadequate closure during systole [7]. Rheumatic MR diagnosis, in particular, requires at least two characteristic morphologies of rheumatic valvulopathies and the four criteria of pathological MR (detailed in Figure 4) [2,6,7]. In the case presented, the TEE showed severe MR with a thickened MV with diastolic doming of the anterior MV leaflet, which is characteristic of RHD. It is important to note that the regurgitating jet size and length do not necessarily correlate with MR severity [7]. MR severity depends on factors such as the vena contracta (VC), the narrowest part of a jet close to the regurgitant orifice, and related to its size [7,13]. Generally, the larger the orifice, the wider the VC, and the greater the regurgitation. Severe MR criteria are summarized in Figure 5 [2,4,6,7]. Other findings of severe MR include LA enlargement, LV dysfunction, and P-HTN [4].

Management

Management of RHD involves the prevention of disease progression and treatment of complications. Primary ARF prophylaxis is focused on early treatment of GAS throat or skin infections before they can trigger the immunological reactions that lead to ARF [2-4,8,9]. Treatment options include a dose of BPG 1.2 million units (900 mg) administered intramuscularly. Alternatively, an oral penicillin or aminopenicillin for 10 days can be used. Secondary prevention involves continuous antibiotics to prevent recurrent GAS infections causing ARF and suppress inflammation [2,4-6,9,14]. A 2021 randomized controlled trial of secondary antibiotic prophylaxis in Ugandan children and adolescents with latent RHD showed a 90% delay in disease progression at two years [6,15]. Intramuscular BPG of 1.2 million units every three to four weeks is preferred over oral penicillin for continuous antimicrobial prophylaxis [2,5,8]. Treatment duration is based on age at ARF diagnosis and RHD severity (summarized in Table 1) [3,5,6]. It is important to note that although the risk of ARF recurrence at ages 40 and beyond is low, lifelong secondary prophylaxis is suggested for patients with severe valvular disease (e.g., leading to heart failure or needing surgery), as seen in this case [4,16].

**Table 1:** Recommended antibiotic prophylaxis duration in different scenarios ARF: acute rheumatic fever. Source [3,5,6,16].

Scenario	Antibiotic prophylaxis duration
Patients with a history of ARF and valvulitis but without residual valvular disease	10 years after the last ARF attack or until age 21, whichever is longer
Patients with persistent valvular disease	10 years after the last attack or until age 40, whichever is longer
Patients younger than 35 with no history of ARF	Five years or until age 40, whichever is longer
Patients with severe valvular injury	Lifelong

Surgical Procedures

The definite treatment of primary MR (as in rheumatic MR) is valve repair or replacement. The 2020 American College of Cardiology (ACC) and American Heart Association (AHA) Guidelines for the Management of Patients with Valvular Heart Disease recommend MV intervention before the onset of LV systolic dysfunction. Ideally, surgery should be performed when the LVEF is 60% or less or when the LV end-systolic diameter is equal to or less than 40 mm [17]. A higher EF cutoff is used due to inherent reduced LV contractility in primary MR, which can affect surgical outcomes. When these parameters are present, patients have already developed LV systolic dysfunction [17,18]. Patients with LV dysfunction or P-HTN have a worse prognosis. Important to note is that when patients become symptomatic, they also have a worse prognosis even when the LV function is preserved. Therefore, patients who are already symptomatic have an indication for timely MV intervention [17]. For these reasons, the patient in our case underwent surgery promptly. Valve repair is generally recommended over valve replacement due to superior survival rates and outcomes. MV repair allows the preservation of the subvalvular apparatus, which favors the conservation of LV shape and contractility [19]. However, the morphological changes and valvular damage in rheumatic MV disease make it less suitable for MV repair. Therefore, MV repair is reserved for less advanced disease cases in which a durable repair can be achieved [17]. The decision between mechanical and bioprosthetic valve replacement depends on several factors, including the patient’s age and preferences, valve durability, and need for anticoagulation, among others. The estimated 15-year risk of reoperation due to bioprosthetic valve degeneration in patients 50, 40, and 30 years of age is 22%, 30%, and 50%, respectively. Therefore, mechanical valve replacement is generally recommended for younger patients [17]. On the other hand, the bleeding risk due to anticoagulation after mechanical valve replacement is higher in older patients, whereas the estimated risk of bioprosthetic valve deterioration in older patients (particularly >65 years) is around 10%. Given that bioprosthetic valve durability is usually greater than life expectancy in patients >65 years, and considering the bleeding risks of anticoagulation, bioprosthetic valve replacement is generally recommended in older patients and those with multiple comorbidities [17]. After shared decision-making in our case, it was determined that a bioprosthetic valve replacement was the most adequate option, considering the patient's age, comorbidities, and her preference to avoid coagulation monitoring.

Atrial Fibrillation

One of the RHD complications is AF, which is associated with poor prognosis. Risk factors include older age, higher LA pressure and lower strain, larger LA size, and lower EF [4]. AF poses a risk of thromboembolic stroke that increases depending on the patient’s age, sex, history of stroke, and other comorbidities. Guidelines recommend using the CHA2DS2-VASc score for risk stratification of thromboembolic stroke to identify suitable candidates for anticoagulation. Patients with AF can be given a direct oral anticoagulant (DOAC), except those with rheumatic MS or mechanical valve replacement, which should be anticoagulated with a vitamin K antagonist [20]. In this case, the patient had a high risk of thromboembolic stroke as per a CHA2DS2-VASc score of 6 (i.e., 9.7% risk of stroke per year) and no contraindications to anticoagulation; therefore, she was started on Apixaban 5 mg twice a day. For patients requiring anticoagulation who undergo simultaneous coronary artery bypass graft and valve surgery, low-dose aspirin is recommended for three months following the procedure, in addition to anticoagulation, to ensure graft patency [21].

Regarding rhythm control, electrical and pharmacological cardioversion are both suitable strategies in hemodynamically stable patients with AF. The efficacy of electrical cardioversion is generally greater than pharmacological cardioversion alone [20]. The patient, in our case, received intravenous amiodarone, which effectively returned the rhythm to normal sinus. The AHA guidelines for AF management state that intravenous amiodarone is a reasonable option for pharmacological cardioversion. It is a first-line antiarrhythmic for the maintenance of normal sinus rhythm in patients with AF and prior myocardial infarction or significant structural heart disease, including heart failure with reduced ejection fraction (HFrEF). Most of the other available antiarrhythmics (except for dofetilide) can worsen heart failure and increase mortality [20]. Amiodarone should be given at the lowest effective dose, and side effects should be monitored, especially in patients with preexisting lung disease, like in the case presented [20]. For rate control of AF, the patient in our case received metoprolol succinate after the resolution of acute heart failure.

Congestive Heart Failure

Another common complication of RHD is heart failure. Guideline-directed therapy (GDMT) for HFrEF is recommended for symptomatic patients with primary MR (as in rheumatic MR) and an LVEF <60%. As mentioned earlier, a higher EF cutoff is used, given the inherent reduced LV contractility in primary MR [17,18]. In general, the GDMT includes medications that inhibit the renin-angiotensin-aldosterone system, beta-blockers, mineralocorticoid receptor antagonists, and sodium-glucose cotransporter-2 (SGLT-2) inhibitors. The beta blockers shown to reduce mortality and hospitalizations in HFpEF are metoprolol succinate, bisoprolol, and carvedilol [22]. Medications like loop diuretics reduce pre-load and are effective for managing symptoms of edema [4,22]. Given primary MR and an LVEF of 50%, the patient in our case was treated with GDMT for HFrEF using valsartan, metoprolol succinate, empagliflozin, and furosemide for edema. As ischemic heart disease is the most common cause of heart failure, the patient underwent a left heart catheterization, which evidenced obstructive coronary artery disease. The etiology of HFpEF in this case is multifactorial, including ischemic heart disease and severe valvulopathy, exacerbated by AF de novo.

## Conclusions

RHD is the leading cause of valvular disease worldwide, mainly affecting MV. Echocardiography is a valuable tool in early diagnosis and severity assessment. Surgery should be performed when the LVEF is 60% or less or when the LV end-systolic diameter is equal to or less than 40 mm. Valve repair is generally recommended over valve replacement, but the morphological changes in rheumatic MV disease make it less suitable for MV repair. The patient’s age and preferences, valve durability, and need for anticoagulation are some of the factors considered to guide the decision between mechanical and bioprosthetic valve replacement. AF and heart failure are common complications of RHD. Patients with AF should be stratified using CHA2DS2-VASc to identify suitable candidates for anticoagulation. Treatment strategies include rate and rhythm control. In patients with AF and significant structural heart disease, amiodarone is a first-line antiarrhythmic for the maintenance of normal sinus rhythm. Patients with primary MR who develop heart failure and have an LVEF <60% should be treated with GDMT for HFrEF. For patients with severe valvular disease, lifelong secondary prophylaxis is recommended.
